# 5-Cyclo­hexyl-1,3-diphenyl-1,3,5-di­aza­phosphinane, its phosphine oxide, and its [NiCl_2_*L*_2_] complex

**DOI:** 10.1107/S2056989025010011

**Published:** 2025-12-14

**Authors:** David Esjornson, Denise Anderson, Jessica Vo, Jeanette A. Krause, Timothy J. Hubin, Allen G. Oliver

**Affiliations:** a Department of Chemistry and Physics Southwestern Oklahoma State University,Weatherford OK 73096 USA; b Department of Chemistry, University of Cincinnati, Cincinnati, OH 45221, USA; c Department of Chemistry and Biochemistry, University of Notre Dame, Notre Dame, IN 46556, USA; University of Missouri-Columbia, USA

**Keywords:** crystal structure, phosphazine, nickel complex

## Abstract

The crystal structures of 5-cyclo­hexyl-1,3-diphenyl-1,3,5-di­aza­phosphinane, C_21_H_27_N_2_P, and its oxidized phosphine oxide, 5-cyclo­hexyl-1,3-diphenyl-1,3,5-di­aza­phosphinan-5-one, C_21_H_27_N_2_OP, have primitive monoclinic symmetry (both in space group *P*2_1_/m) at 150 K. The nickel(II) complex *trans*-di­chlorido­bis­(5-cyclo­hexyl-1,3-diphenyl-1,3,5-di­aza­phosphinane-κ*P*)nickel(II), [NiCl_2_(C_21_H_27_N_2_P)_2_], consists of two di­aza­phosphinane ligands (monoclinic *C*2/*c*, 150 K) bound through their phospho­rous atoms, which adopts a four-coordinate square-planar geometry. The bulky cyclo­hexyl substituents of the ligand are axially positioned in their respective chair six-membered ligand rings, and are in an *anti*-configuration, with respect to the square plane. The nickel atom is located on a center of symmetry.

## Abstract 2

Crystal structures have been obtained for the heterocyclic NPN compound, 5-cyclo­hexyl-1,3-diphenyl-1,3,5-di­aza­phosphinane and its air oxidized phosphine oxide, 5-cyclo­hexyl-1,3-diphenyl-1,3,5-di­aza­phosphinan-5-one. The nickel(II) dichloride complex with bis di­aza­phosphinane ligands has also been obtained and structurally characterized.

## 1. Chemical context

Tri-substituted six-membered NPN heterocycles are known and generally require a substituent on the phospho­rous atom for stability. The best characterized is 1,3,5-triphenyl-1,3,5-di­aza­phosphinane, which has been known since 1979 (Arbuzov *et al.*, 1979 ▸). Relevant papers detail the original synthesis (Arbuzov *et al.*, 1979 ▸), an additional synthetic route (Maerkl & Yu, 1981 ▸), its oxidation to the phosphine oxide (Arbuzov *et al.*, 1980 ▸), axial/equatorial conformational equilibria of its phenyl substitutents (Arbuzov *et al.*, 1981 ▸), and its complexation with transition-metal ions (Karasik *et al.*, 1993 ▸, 1996*a* ▸,*b* ▸; Khadiullin *et al.*, 1993 ▸; Pisarevskii *et al.*, 1995 ▸). An additional six-membered NPN ligand, 1,3-di­cyclo­hexyl-5-phenyl-1,3,5-di­aza­phosphinane, has been reported (Karsch *et al.*, 1997 ▸) with two cyclo­hexyl groups on the two nitro­gen atoms, and a phenyl substituent on the phospho­rous. However, it is not structurally characterized.

In an attempt to diversify this family of compounds, we were able to produce a new six-membered NPN ligand, 5-cyclo­hexyl-1,3-diphenyl-1,3,5-di­aza­phosphinane (**I**). We found that the phosphine was air sensitive in the presence of nickel(II) and air, and it could be oxidized to its phosphine oxide (**II**), which has also been structurally characterized. However, further attempts to produce this phosphine oxide by independent, intentional air oxidation of (**I**) have not been successful in our hands. The original phosphine was deemed likely to be a good ligand for transition metals, as evidenced by the other members of the ligand family (Karasik *et al.*, 1993 ▸, 1996*a* ▸,*b* ▸; Khadiullin *et al.*, 1993 ▸). We successfully produced a *trans*, square-planar nickel(II) complex containing two of the ligands, compound (**III**). The structural details of these compounds will be disclosed and discussed below.
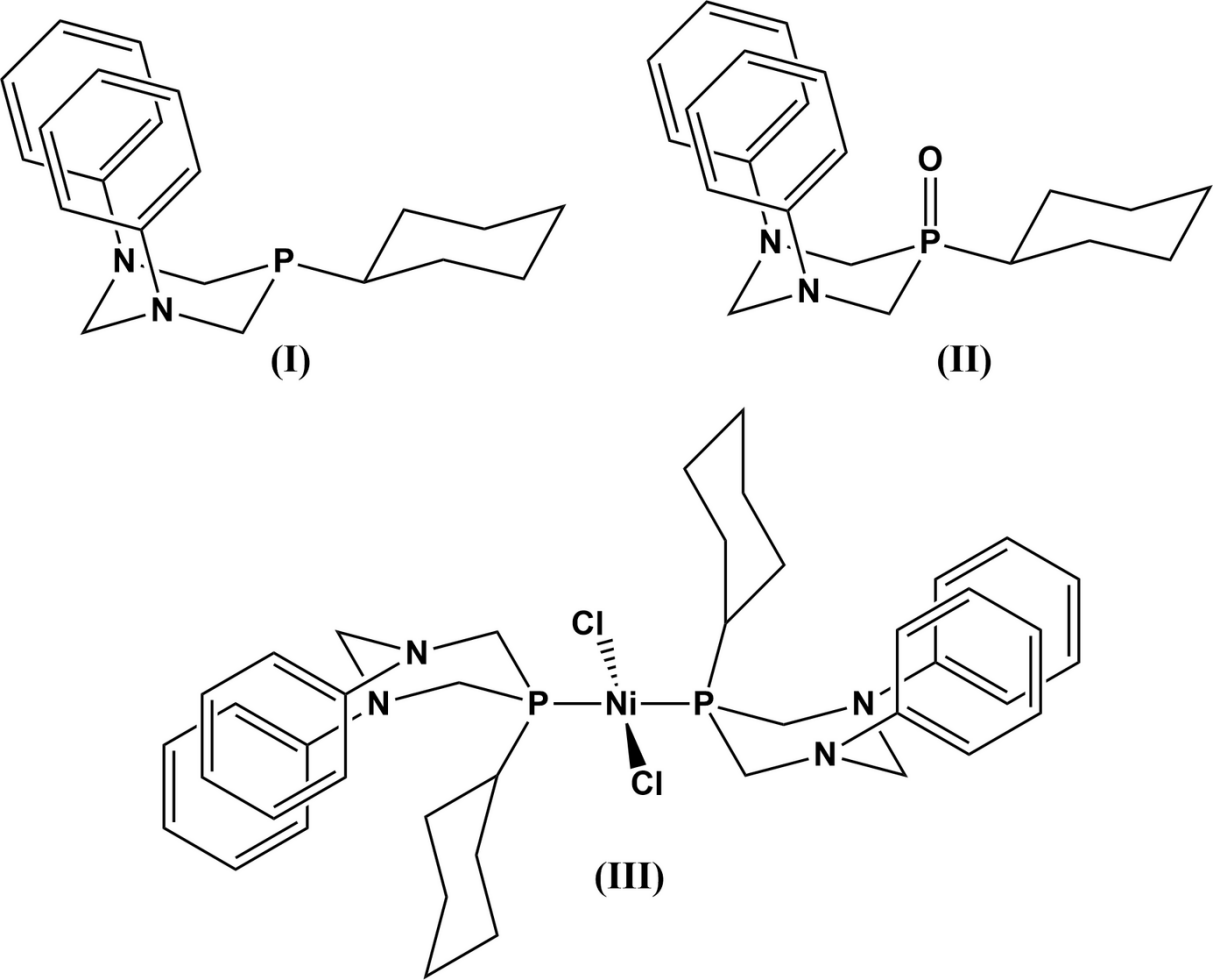


## 2. Structural commentary

**Figure 1:**
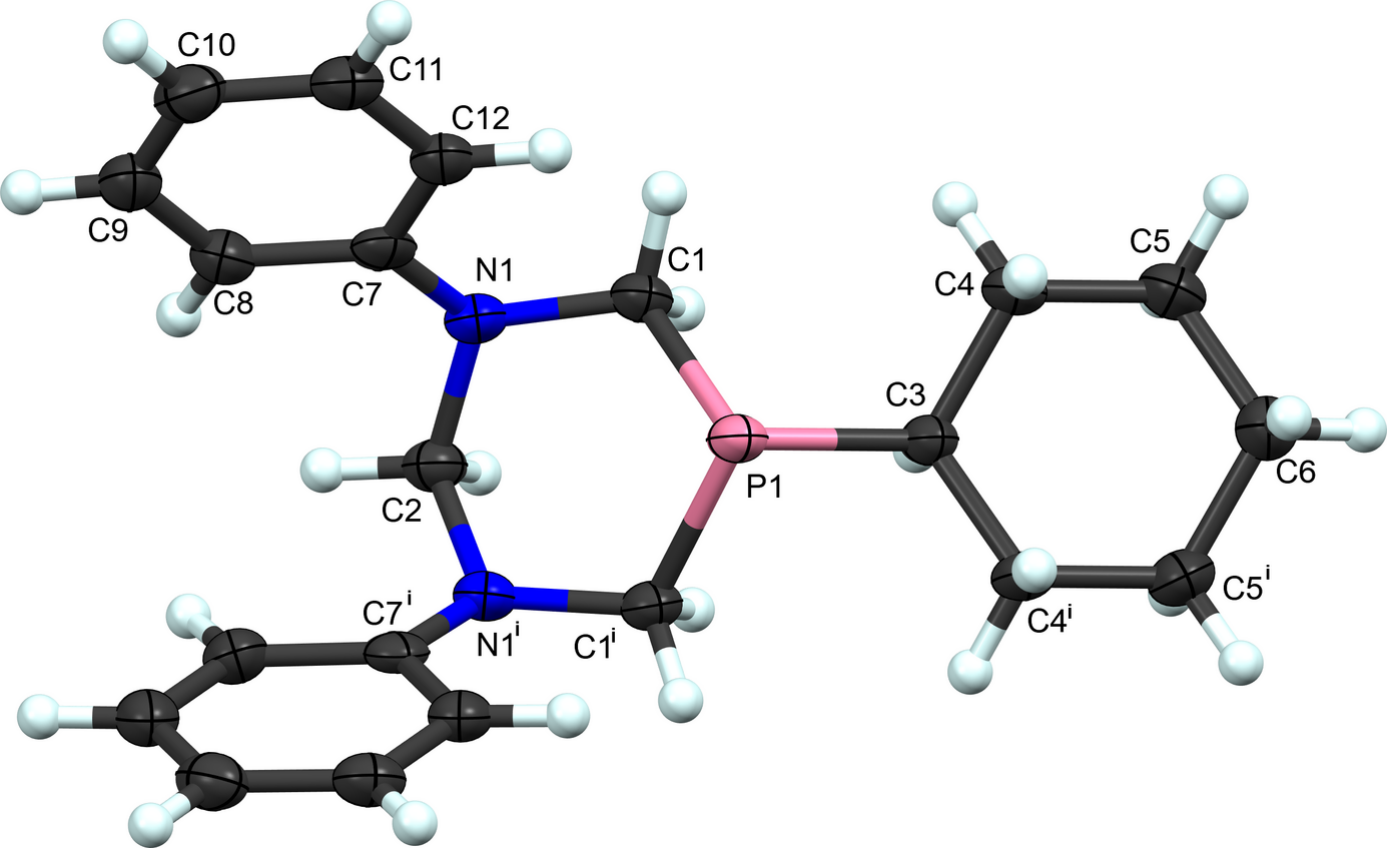
The labeling scheme for 5-cyclo­hexyl-1,3-diphenyl-1,3,5-di­aza­phosphinane (**I**). Atomic displacement ellipsoids shown at 50% probability and hydrogen atoms as spheres of an arbitrary radius. Symmetry code: (i) *x*, −*y* + 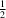, *z.*

5-Cyclo­hexyl-1,3-diphenyl-1,3,5-di­aza­phosphinane, (**I**) (Fig. 1 ▸), is a rare example of the ligand-only structurally characterized six-membered NPN heterocycle. Most of the similar known di­aza­phosphinane ligands also incorporate benzyl or larger substituents bonded to the N and P atoms. Its main ring and the cyclo­hexyl group are both found in chair conformations with each ring equatorially located on the other. The unique phenyl group is oriented in an axial fashion on the nitro­gen atom. The mol­ecule is located on the crystallographic mirror plane at (*x*, 0.25, *z*) with C2, P1, C3 and C6 located on the plane. The bond angles about the phospho­rus are all smaller than an ideal tetra­hedral angle (Table 1 ▸). In contrast, the angles about the unique nitro­gen atom are more relaxed and tend to a more obtuse angle (Table 1 ▸).

**Figure 2:**
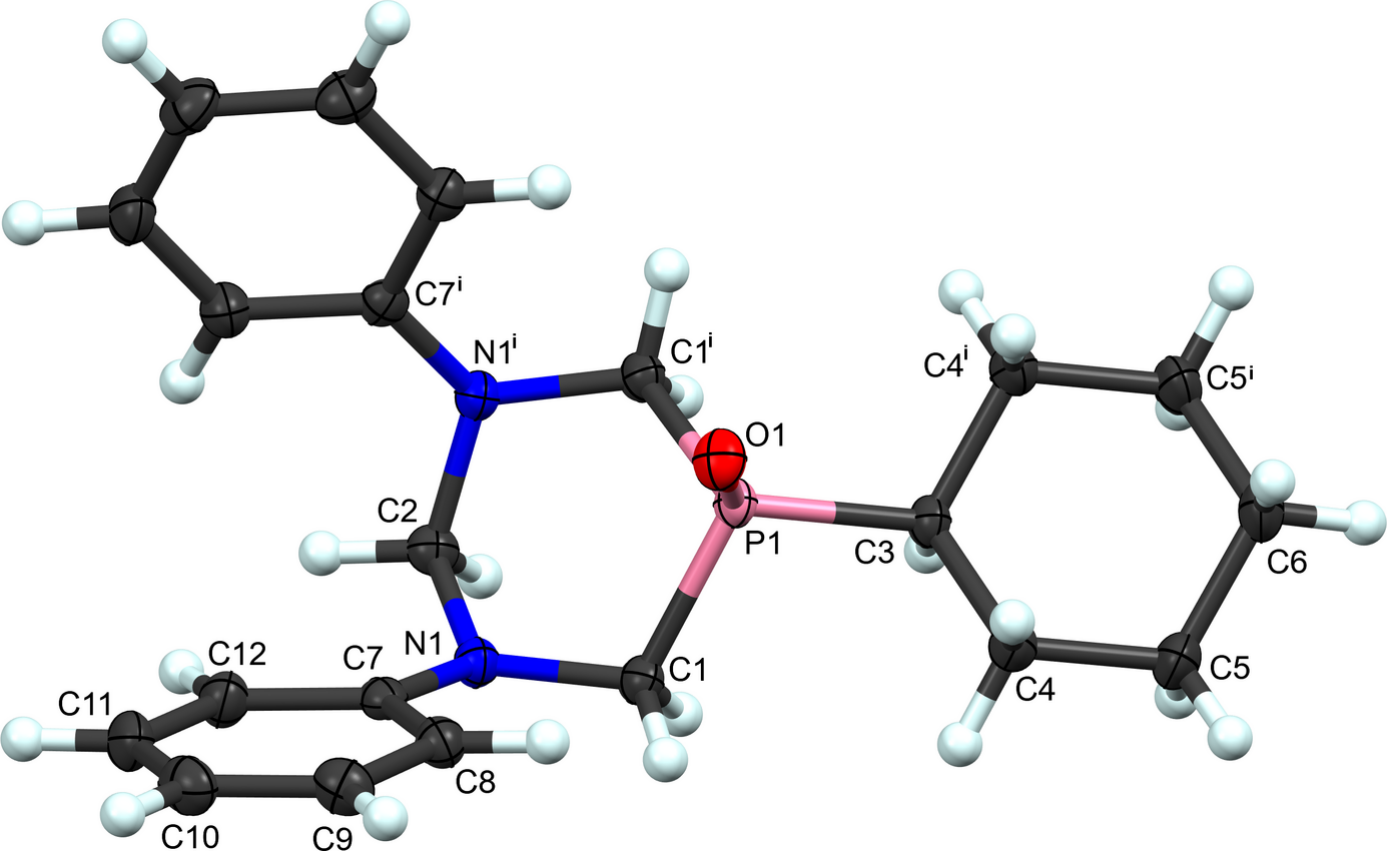
The labeling scheme for 5-cyclo­hexyl-1,3-diphenyl-1,3,5-di­aza­phosphinan-5-one (**II**). Atomic displacement ellipsoids shown at 50% probability and hydrogen atoms as spheres of an arbitrary radius. Symmetry code: (i) *x*, −*y* + 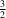, *z.*

5-Cyclo­hexyl-1,3-diphenyl-1,3,5-di­aza­phosphinan-5-one, (**II**) (Fig. 2 ▸), maintains much of the same geometry, and can be described in similar terms, except for the added P=O double bond. It too resides on the mirror plane at (*x*, 0.75, *z*) that bis­ects the mol­ecule through C2, P1, O1, C3, and C6. The bond angles about the phospho­rus atom have increased compared with those in the unoxidized form (Table 2 ▸). Surprisingly, the C1—P—C1^i^ angle is still more acute than an ideal tetra­hedral angle, although it has changed significantly compared with the parent (**I**) [symmetry code: (i) *x*, −*y* + 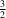, *z*].

**Figure 3:**
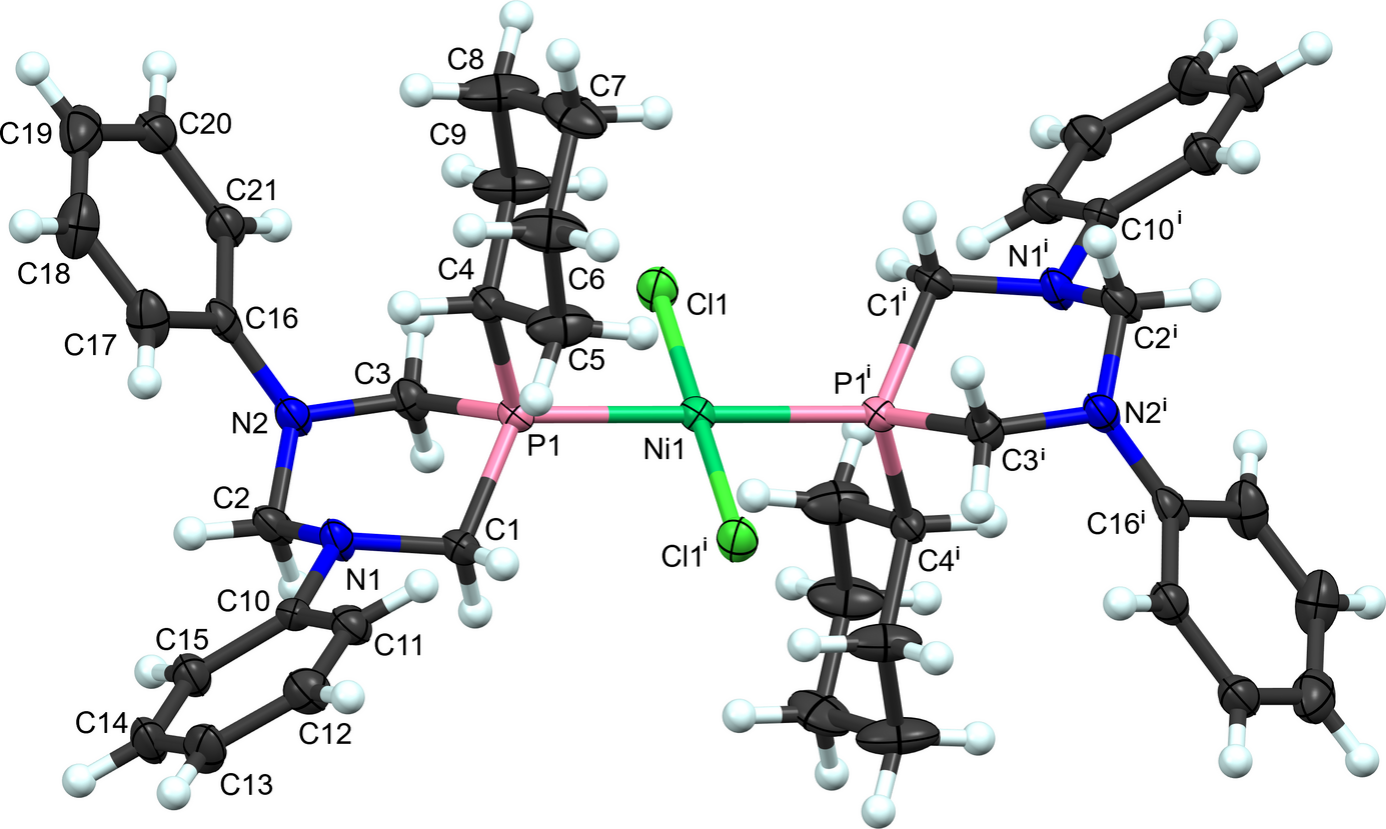
The labeling scheme for the complex *trans*-di­chloro­bis­(5-cyclo­hexyl-1,3-diphenyl-1,3,5-di­aza­phosphinan-5-yl)nickel(II) (**III**). Atomic displacement ellipsoids shown at 50% probability and hydrogen atoms as spheres of an arbitrary radius. Symmetry code: (i) −*x* + 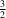, −*y* + 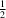, −*z* + 1.

The coordination complex, *trans*-di­chloro-bis­(5-cyclo­hexyl-1,3-diphenyl-1,3,5-di­aza­phosphinan-5-yl)-nickel(II), (**III**) (Fig. 3 ▸), contains a square-planar nickel(II) ion with two *trans* 5-cyclo­hexyl-1,3-diphenyl-1,3,5-di­aza­phosphinane (I) ligands, coordinated *via* the phospho­rus atom and two *trans* chloro ligands. The nickel atom is located on a center of symmetry (0.75, 0.25, 0.5). The coordination geometry around nickel is nearly perfectly square planar, with all *cis*-bond angles close to 90° (Table 3 ▸). Notably, the six-membered heterocyclic ring is ring-flipped compared with the free ligand structure, because the cyclo­hexyl substituent is now in an axial position (rather than equatorial in the free ligand) and the phenyl substituents are in equatorial positions (rather than axial in the free ligand). Axial/equatorial conformational equilibria of its phenyl substituents in related NPN heterocycle 1,3,5-triphenyl-1,3,5-di­aza­phosphinane have previously been described (Arbuzov *et al.*, 1981 ▸). As noted below in the *Database survey*, similar triphenyl ligands are able to bind in a *cis* fashion to Mo^0^, Pt^II^, and Pd^II^ (Karasik *et al.*, 1993 ▸, 1996*b* ▸; Pisarevskii *et al.*, 1995 ▸). The bulkier cyclo­hexyl substituent on the phospho­rous atom likely contributes to the need for *trans* coordination in the case of (**III**). Additionally, this steric bulk requires an axial orientation of the cyclo­hexyl group in order to coordinate to the nickel(II) center. Finally, the two cyclo­hexyl groups from the two heterocyclic ligands are found in a necessarily *anti*-configuration about the square plane, enforced by the center of symmetry, and likely due to their steric bulk.

## 3. Supra­molecular features

The packing of compound (**I**) is solely influenced by van de Waals inter­actions. The lack of directional electropositive coupled with electronegative elements in the structure enforces this. Although (**II**) has been oxidized and includes a potential hydrogen-bond acceptor (O1), there are no hydrogen-bond donor atoms in the mol­ecule. Thus, the only inter­molecular inter­actions for (**II**) are through van der Waals contacts. Compounds (**I**) and (**II**) are essentially isostructural with very similar cell parameters (Table 4 ▸) and an identical packing motif, despite the presence of the additional oxygen atom in (**II**). Similarly, despite being coordinated to a metal center that also contains chlorine atoms, compound (**III**) contains no strong, electropositive groups and the extended structure is again dictated by van der Waals inter­actions.

## 4. Database survey

No structures of the published similar ligands, 1,3,5-triphenyl-1,3,5-di­aza­phosphinane and 1,3-di­cyclo­hexyl-5-phenyl-1,3,5-di­aza­phosphinane, nor of their phosphine oxides were found in the CSD v2025.2.0, Aug 2025 update; Groom *et al.*, 2016 ▸). However, four different transition-metal complexes of 1,3,5-triphenyl-1,3,5-di­aza­phosphinane have been deposited CSD refcodes [TECZAC (Karasik *et al.*, 1996*b* ▸), YUXNEK (Pisarevskii *et al.*, 1995 ▸), YUXKEH (Karasik *et al.*, 1993 ▸), and YUXKAD (Karasik *et al.*, 1993 ▸)]. Three of these complexes are four-coordinate complexes with two of the bulky 1,3,5-triphenyl-1,3,5-di­aza­phosphinane ligand bound surprisingly, in a *cis*-square-planar geometry to di­chloro­palladium(II) (YUXNEK) and di­chloro­platinum(II) (two different crystal forms: one is unsolvated in the solid state, the second is an aceto­nitrile/water solvate; YUXKAD, YUXKEH). The fourth reported structure containing 1,3,5-triphenyl,-1,3,5-di­aza­phosphinane ligand is an octa­hedral molybdenum tetra­carbonyl complex (TECZAC). The bulky phosphinane ligands adopt a very similar inter­nal conformation and ligand/ligand arrangement around the molybdenum, similar to the palladium complex. The steric bulk of the phenyl groups on the two phospho­rous atoms of the separate ligands can be accommodated in a *cis* arrangement around the respective metal ion, in both square-planar and octa­hedral coordination geometries. This *cis* arrangement contrasts with the *trans* arrangement of *trans*-di­chloro-bis­(5-cyclo­hexyl-1,3-diphenyl-1,3,5-di­aza­phosphin­an-5-yl)nickel(II) in structure (**III**) (Fig. 3 ▸). The steric bulk of the cyclo­hexyl substituent in this new ligand is greater than that of a phenyl group, and perhaps this bulk is enough to drive the formation of the *trans* nickel(II) complex. This may be a useful property of this new ligand if *trans* complexes are desired.

## 5. Synthesis and crystallization

**Preparation of the precursor cyclo­hexyl­bis­(phenyl­amino­meth­yl)phosphine**

In an inert atmosphere glovebox, to cyclo­hexyl­phosphine (10.00 g, 0.0861 mol) in 100 mL of ethanol was added para­formaldehyde (5.20 g, 0.399 mol). A white suspension formed and was left to stir. After 3 d, the solution was removed from the glovebox. In a separate container, 25 mL of aniline was mixed with 80 mL of ethanol and heated to reflux. The hot aniline/ethanol solution was dripped into the cyclo­hexyl­phosphine solution over 30 min and the whole solution refluxed for 1 h and left to cool while stirring. After 3 d, a white precipitate was filtered from the solution and washed with 125 mL ethanol. The filtrate was evaporated at 323 K to dryness and placed under vacuum for 20 min. To the reduced solution were added 20 mL of pentane and the flask allowed to sit for 2 d at room temperature. The filtrate contained excess aniline, which dissolved into the pentane, and two layers formed. Crystals of di­amino phosphine formed in the bottom layer. The liquid was deca­nted off, and the crystallized layer was filtered and washed with minimal amounts of pentane. The precipitate yielded 10.164 g of the phosphine (36% yield).
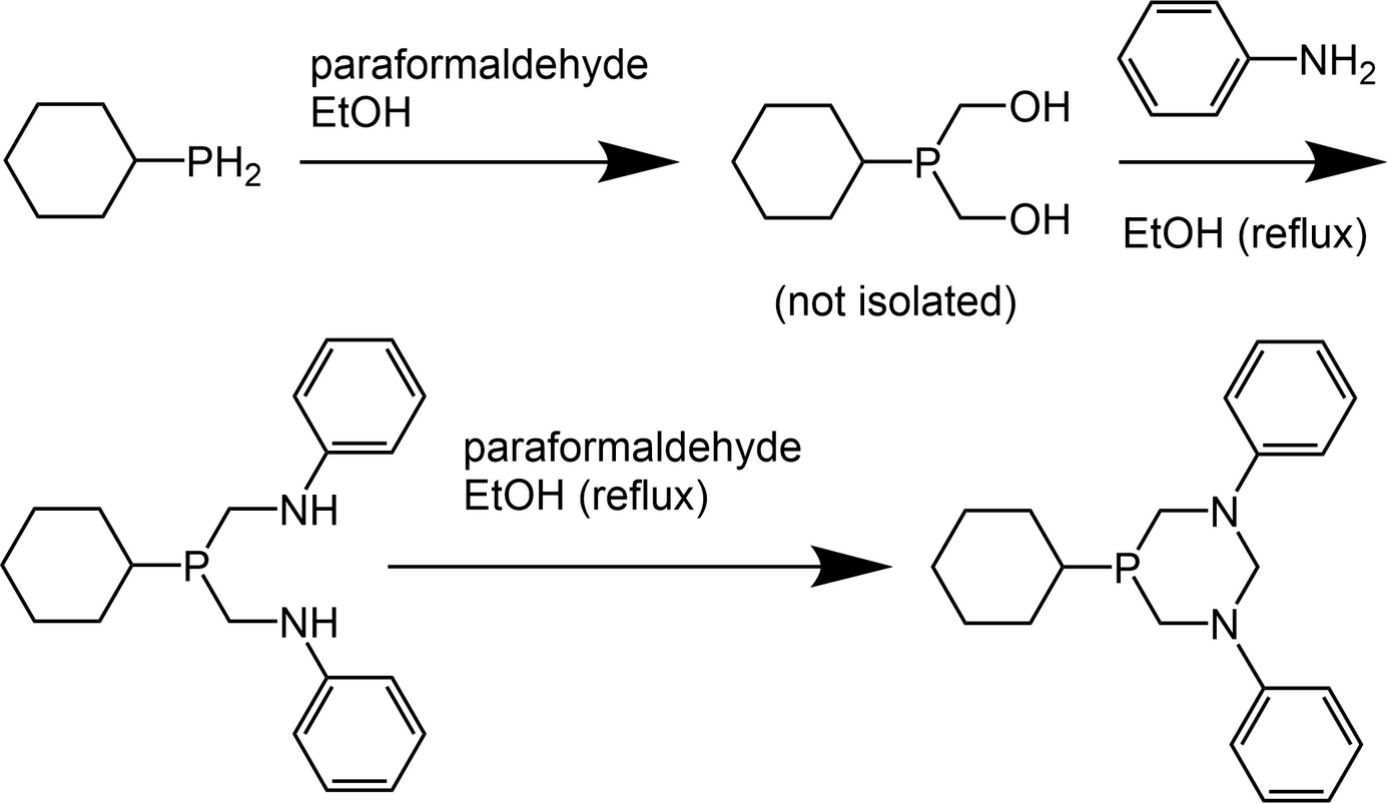


**Preparation of 5-cyclo­hexyl-1,3-diphenyl-1,3-di­aza-5-phospha­cyclo­hexane (I)**

In an inert atmosphere glovebox, paraformaldehyde (5.00 g, 0.384 mol) was mixed with cyclo­hexyl­bis­(phenyl­amino­meth­yl)phosphine (10.4 g, 0.0861 mol) in 100 mL of ethanol and left to stir for 3 d. The solution was removed from the glovebox. In a separate container, 25 mL aniline (0.276 mol) in 80 mL of ethanol (1.370 mol) was heated to reflux. The cyclo­hexyl­phosphine solution was slowly dripped into the heated solution. An additional 75 mL ethanol was used to rinse the cyclo­hexyl­phosphine flask and added to the aniline solution. The solution was refluxed for 1 h then left to cool and stir for 3 d. A white precipitate was filtered and rinsed with 100 mL ethanol. The precipitate was washed with diethyl ether and dried under vacuum to give 6.495 g (22% yield) of the final product (**I**). X-ray quality crystals were obtained by di­chloro­methane diffusion into an ethanol solution.

**Preparation of 5-cyclo­hexyl-1,3-diphenyl-1,3,5-di­aza­phosphinan-5-one (II), and** ***trans*** **-di­chloro-bis­(5-cyclo­hexyl-1,3-diphenyl-1,3,5-di­aza­phosphinan-5-yl)-nickel(II) (III)**

To a solution of 5-cyclo­hexyl-1,3-diphenyl-1,3-di­aza-5-phospha­cyclo­hexane (0.163 g, 0.48 mmol) in 10 mL of DMF was added nickel(II) chloride (0.065 g, 0.50 mmol). The solution was left to stir for 4 d open to the air. After stirring, the solution was filtered, and the precipitate was washed with minimal amounts of DMF followed by diethyl ether. The combined washings and filtrate were reduced to 1/3 volume under reduced pressure. The additional precipitate was filtered and washed with minimal amounts of diethyl ether, giving a total of 0.159 g of total product (70% yield). Two types of crystals were obtained from the precipitated material. Blue–green plates obtained from the mixture were structurally characterized as the nickel complex (**III**), and colorless plates were structurally characterized as the phosphine oxide (**II**). Phosphine oxide (**II**) has not been produced by any other method in our hands.

## 6. Refinement

Data were recorded on Advanced Light Source beamlines 11.3.1 (**I**) or 12.2.1 (**II**), (**III**) with a Bruker Photon-100 or Bruker Photon-II detector, respectively (Bruker, 2019 ▸). It should be noted that the sample sizes range from 0.01 to 0.06 mm (10 to 60 micrometers) and it was particularly challenging to find samples suitable for diffraction. Hence access to a synchrotron source was required to measure these crystals. Some artifacts from the measurement do appear in the data (slightly higher *R*_int_ values for example). However, the models are still suitable and correct. Data analysis followed a routine workflow for corrections and space group analysis. All three structures were solved using dual-space methods (Sheldrick, 2015*a* ▸) and refined routinely (Sheldrick, 2015*b* ▸, Table 4 ▸). Anomalous scattering and mass attenuation factors appropriate for the wavelengths accessed at the two sources were determined by Brennan & Cowan (1992 ▸) methods, in *PLATON* (Spek, 2020 ▸). Non-hydrogen atoms were treated with an anisotropic model and hydrogen atoms were included in calculated positions, riding on the atoms to which they are bonded with *U*_iso_(H) = 1.2 × *U*_eq_(C).

## Supplementary Material

Crystal structure: contains datablock(s) I, II, III. DOI: 10.1107/S2056989025010011/ev2023sup1.cif

Structure factors: contains datablock(s) I. DOI: 10.1107/S2056989025010011/ev2023Isup2.hkl

Structure factors: contains datablock(s) II. DOI: 10.1107/S2056989025010011/ev2023IIsup3.hkl

Structure factors: contains datablock(s) III. DOI: 10.1107/S2056989025010011/ev2023IIIsup4.hkl

Supporting information file. DOI: 10.1107/S2056989025010011/ev2023Isup5.cml

Supporting information file. DOI: 10.1107/S2056989025010011/ev2023IIsup6.cml

CCDC references: 2501742, 2501741, 2501740

Additional supporting information:  crystallographic information; 3D view; checkCIF report

## Figures and Tables

**Table 1:** Selected geometric parameters (Å, °) for (**I**)

P1—C3	1.862 (3)	N1—C7	1.403 (3)
P1—C1^i^	1.888 (2)	N1—C2	1.466 (3)
P1—C1	1.888 (2)	N1—C1	1.470 (3)
			
C3—P1—C1^i^	98.94 (11)	C7—N1—C2	120.8 (2)
C3—P1—C1	98.94 (11)	C7—N1—C1	120.5 (2)
C1^i^—P1—C1	92.49 (16)	C2—N1—C1	111.7 (2)

Symmetry code: (i) 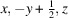.

**Table 2:** Selected geometric parameters (Å, °) for (**II**)

P1—O1	1.4924 (13)	N1—C7	1.3999 (15)
P1—C3	1.8114 (17)	N1—C1	1.4609 (15)
P1—C1^i^	1.8309 (12)	N1—C2	1.4615 (14)
P1—C1	1.8309 (12)		
			
O1—P1—C3	114.82 (8)	C1^i^—P1—C1	98.38 (8)
O1—P1—C1^i^	115.28 (5)	C7—N1—C1	121.28 (10)
C3—P1—C1^i^	105.65 (5)	C7—N1—C2	121.71 (11)
O1—P1—C1	115.28 (5)	C1—N1—C2	111.95 (11)
C3—P1—C1	105.65 (5)		

Symmetry code: (i) 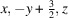.

**Table 3:** Selected geometric parameters (Å, °) for (**III**)

Ni1—Cl1^i^	2.1736 (8)	P1—C1	1.829 (3)
Ni1—Cl1	2.1736 (8)	P1—C3	1.841 (3)
Ni1—P1	2.2138 (8)	P1—C4	1.841 (3)
Ni1—P1^i^	2.2138 (8)		
			
Cl1^i^—Ni1—Cl1	180.0	C1—P1—C3	97.82 (14)
Cl1^i^—Ni1—P1	90.03 (3)	C1—P1—C4	105.16 (14)
Cl1—Ni1—P1	89.97 (3)	C3—P1—C4	108.38 (14)
Cl1^i^—Ni1—P1^i^	89.97 (3)	C1—P1—Ni1	116.88 (10)
Cl1—Ni1—P1^i^	90.03 (3)	C3—P1—Ni1	115.44 (10)
P1—Ni1—P1^i^	180.0	C4—P1—Ni1	111.83 (10)

Symmetry code: (i) 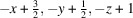.

**Table 4:** Experimental details

	(**I**)	(**II**)	(**III**)
Crystal data			
Chemical formula	C_21_H_27_N_2_P	C_21_H_27_N_2_OP	[NiCl_2_(C_21_H_27_N_2_P)_2_]
*M*_r_	338.41	354.41	806.44
Crystal system, space group	Monoclinic, *P*2_1_/*m*	Monoclinic, *P*2_1_/*m*	Monoclinic, *C*2/*c*
Temperature (K)	150	150	150
*a*, *b*, *c* (Å)	5.3767 (17), 14.082 (4), 12.165 (4)	5.2996 (3), 14.1195 (7), 12.0711 (6)	23.5711 (15), 10.0062 (6), 17.8428 (10)
β (°)	98.560 (6)	96.736 (2)	109.371 (3)
*V* (Å^3^)	910.8 (5)	897.02 (8)	3970.1 (4)
*Z*	2	2	4
Radiation type	Synchrotron, λ = 1.0333 Å	Synchrotron, λ = 0.7288 Å	Synchrotron, λ = 0.7288 Å
μ (mm^−1^)	0.42	0.17	0.79
Crystal size (mm)	0.08 × 0.04 × 0.02	0.04 × 0.03 × 0.01	0.06 × 0.04 × 0.04

Data collection			
Diffractometer	Bruker D8	Bruker D8	Bruker D8
Absorption correction	Multi-scan (*SADABS*; Krause *et al.*, 2015 ▸)	Multi-scan (*SADABS*; Krause *et al.*, 2015 ▸)	Multi-scan (*SADABS*; Krause *et al.*, 2015 ▸)
*T*_min_, *T*_max_	0.508, 0.748	0.696, 0.746	0.690, 0.746
No. of measured, independent and observed [*I* > 2σ(*I*)] reflections	6147, 1903, 1469	24018, 2313, 1956	36108, 4383, 3341
*R*_int_	0.078	0.047	0.103
(sin θ/λ)_max_ (Å^−1^)	0.624	0.667	0.642

Refinement			
*R*[*F*^2^ > 2σ(*F*^2^)], *wR*(*F*^2^), *S*	0.060, 0.160, 1.11	0.034, 0.087, 1.03	0.056, 0.101, 1.07
No. of reflections	1903	2313	4383
No. of parameters	115	121	232
H-atom treatment	H-atom parameters constrained	H-atom parameters constrained	H-atom parameters constrained
Δρ_max_, Δρ_min_ (e Å^−3^)	0.36, −0.55	0.32, −0.37	0.37, −0.41

Computer programs: *APEX3* and *SAINT* (Bruker, 2019 ▸), *SHELXT2018/2* (Sheldrick, 2015*a* ▸), *SHELXL2019/3* (Sheldrick, 2015*b* ▸), *Mercury* (Macrae *et al.*, 2020 ▸) and *publCIF* (Westrip, 2010 ▸).

## supplementary crystallographic information

### 5-Cyclohexyl-1,3-diphenyl-1,3,5-diazaphosphinane (I) Crystal data

**Table:**

C_21_H_27_N_2_P	*F*(000) = 364
*M**_r_* = 338.41	*D*_x_ = 1.234 Mg m^−^^3^
Monoclinic, *P*2_1_/*m*	Synchrotron radiation, λ = 1.0333 Å
*a* = 5.3767 (17) Å	Cell parameters from 3418 reflections
*b* = 14.082 (4) Å	θ = 3.2–40.0°
*c* = 12.165 (4) Å	µ = 0.42 mm^−^^1^
β = 98.560 (6)°	*T* = 150 K
*V* = 910.8 (5) Å^3^	Tablet, colorless
*Z* = 2	0.08 × 0.04 × 0.02 mm

### 5-Cyclohexyl-1,3-diphenyl-1,3,5-diazaphosphinane (I) Data collection

**Table:**

Bruker D8 diffractometer	1903 independent reflections
Radiation source: synchrotron	1469 reflections with *I* > 2σ(*I*)
Channel-cut Si-<111> monochromator	*R*_int_ = 0.078
Detector resolution: 7.41 pixels mm^-1^	θ_max_ = 40.1°, θ_min_ = 2.5°
combination of ω and φ–scans	*h* = −6→6
Absorption correction: multi-scan (SADABS; Krause *et al.*, 2015)	*k* = −17→17
*T*_min_ = 0.508, *T*_max_ = 0.748	*l* = −14→15
6147 measured reflections	

### 5-Cyclohexyl-1,3-diphenyl-1,3,5-diazaphosphinane (I) Refinement

**Table:**

Refinement on *F*^2^	Primary atom site location: dual
Least-squares matrix: full	Secondary atom site location: difference Fourier map
*R*[*F*^2^ > 2σ(*F*^2^)] = 0.060	Hydrogen site location: inferred from neighbouring sites
*wR*(*F*^2^) = 0.160	H-atom parameters constrained
*S* = 1.11	*w* = 1/[σ^2^(*F*_o_^2^) + (0.0643*P*)^2^ + 0.4286*P*] where *P* = (*F*_o_^2^ + 2*F*_c_^2^)/3
1903 reflections	(Δ/σ)_max_ < 0.001
115 parameters	Δρ_max_ = 0.36 e Å^−^^3^
0 restraints	Δρ_min_ = −0.55 e Å^−^^3^

### 5-Cyclohexyl-1,3-diphenyl-1,3,5-diazaphosphinane (I) Special details

**Table:**

Geometry. All esds (except the esd in the dihedral angle between two l.s. planes) are estimated using the full covariance matrix. The cell esds are taken into account individually in the estimation of esds in distances, angles and torsion angles; correlations between esds in cell parameters are only used when they are defined by crystal symmetry. An approximate (isotropic) treatment of cell esds is used for estimating esds involving l.s. planes.

### 5-Cyclohexyl-1,3-diphenyl-1,3,5-diazaphosphinane (I) Fractional atomic coordinates and isotropic or equivalent isotropic displacement parameters (Å^2^)

**Table:**

	*x*	*y*	*z*	*U*_iso_*/*U*_eq_	
P1	0.39516 (17)	0.250000	0.59171 (7)	0.0293 (3)	
N1	0.5578 (4)	0.33722 (13)	0.40867 (16)	0.0308 (5)	
C1	0.5739 (5)	0.34684 (16)	0.5299 (2)	0.0309 (6)	
H1A	0.505217	0.409371	0.547311	0.037*	
H1B	0.752676	0.344618	0.564214	0.037*	
C2	0.6798 (7)	0.250000	0.3782 (3)	0.0315 (8)	
H2A	0.856867	0.250000	0.414947	0.038*	
H2B	0.680768	0.250001	0.296860	0.038*	
C3	0.5719 (7)	0.250000	0.7355 (3)	0.0300 (8)	
H3A	0.755820	0.250001	0.730465	0.036*	
C4	0.5118 (5)	0.33978 (17)	0.7998 (2)	0.0368 (6)	
H4A	0.557121	0.397018	0.759871	0.044*	
H4B	0.328976	0.342251	0.803012	0.044*	
C5	0.6568 (6)	0.33984 (18)	0.9181 (2)	0.0436 (7)	
H5A	0.608779	0.396533	0.958282	0.052*	
H5B	0.839382	0.343817	0.914757	0.052*	
C6	0.6031 (8)	0.250000	0.9824 (3)	0.0430 (10)	
H6A	0.709754	0.250000	1.056151	0.052*	
H6B	0.424941	0.250000	0.994313	0.052*	
C7	0.3552 (5)	0.37690 (15)	0.3374 (2)	0.0298 (6)	
C8	0.3520 (5)	0.37552 (17)	0.2207 (2)	0.0350 (6)	
H8	0.484137	0.344781	0.190768	0.042*	
C9	0.1591 (5)	0.41829 (18)	0.1498 (2)	0.0380 (6)	
H9	0.158656	0.414859	0.071758	0.046*	
C10	−0.0342 (5)	0.46622 (18)	0.1907 (2)	0.0393 (7)	
H10	−0.164488	0.496323	0.141625	0.047*	
C11	−0.0323 (5)	0.46904 (17)	0.3048 (2)	0.0363 (6)	
H11	−0.162969	0.501599	0.333830	0.044*	
C12	0.1576 (5)	0.42505 (16)	0.3777 (2)	0.0320 (6)	
H12	0.153618	0.427566	0.455505	0.038*	

### 5-Cyclohexyl-1,3-diphenyl-1,3,5-diazaphosphinane (I) Atomic displacement parameters (Å^2^)

**Table:**

	*U*^11^	*U*^22^	*U*^33^	*U*^12^	*U*^13^	*U*^23^
P1	0.0385 (5)	0.0182 (4)	0.0299 (5)	0.000	0.0010 (4)	0.000
N1	0.0412 (12)	0.0194 (10)	0.0317 (12)	0.0000 (8)	0.0051 (9)	0.0015 (8)
C1	0.0407 (14)	0.0192 (11)	0.0320 (14)	−0.0019 (10)	0.0026 (10)	−0.0017 (10)
C2	0.038 (2)	0.0216 (16)	0.035 (2)	0.000	0.0077 (15)	0.000
C3	0.040 (2)	0.0197 (16)	0.0284 (19)	0.000	−0.0013 (14)	0.000
C4	0.0573 (17)	0.0186 (12)	0.0336 (15)	0.0011 (11)	0.0041 (12)	−0.0024 (10)
C5	0.068 (2)	0.0254 (13)	0.0362 (16)	−0.0027 (12)	0.0032 (13)	−0.0059 (11)
C6	0.065 (3)	0.033 (2)	0.031 (2)	0.000	0.0051 (18)	0.000
C7	0.0381 (14)	0.0145 (10)	0.0365 (15)	−0.0056 (9)	0.0043 (11)	0.0005 (9)
C8	0.0467 (15)	0.0243 (12)	0.0341 (15)	0.0008 (11)	0.0063 (11)	−0.0029 (10)
C9	0.0543 (17)	0.0264 (13)	0.0324 (15)	−0.0046 (12)	0.0034 (12)	0.0004 (10)
C10	0.0448 (15)	0.0275 (13)	0.0424 (17)	−0.0015 (11)	−0.0041 (12)	0.0044 (11)
C11	0.0425 (15)	0.0227 (12)	0.0437 (17)	0.0010 (11)	0.0066 (12)	0.0005 (11)
C12	0.0417 (14)	0.0213 (12)	0.0332 (14)	−0.0030 (10)	0.0058 (11)	0.0002 (10)

### 5-Cyclohexyl-1,3-diphenyl-1,3,5-diazaphosphinane (I) Geometric parameters (Å, º)

**Table:**

P1—C3	1.862 (3)	C5—C6	1.537 (3)
P1—C1^i^	1.888 (2)	C5—H5A	0.9900
P1—C1	1.888 (2)	C5—H5B	0.9900
N1—C7	1.403 (3)	C6—H6A	0.9900
N1—C2	1.466 (3)	C6—H6B	0.9900
N1—C1	1.470 (3)	C7—C12	1.409 (4)
C1—H1A	0.9900	C7—C8	1.417 (4)
C1—H1B	0.9900	C8—C9	1.385 (4)
C2—H2A	0.9900	C8—H8	0.9500
C2—H2B	0.9900	C9—C10	1.392 (4)
C3—C4	1.546 (3)	C9—H9	0.9500
C3—C4^i^	1.546 (3)	C10—C11	1.387 (4)
C3—H3A	1.0000	C10—H10	0.9500
C4—C5	1.532 (4)	C11—C12	1.394 (3)
C4—H4A	0.9900	C11—H11	0.9500
C4—H4B	0.9900	C12—H12	0.9500
			
C3—P1—C1^i^	98.94 (11)	C4—C5—C6	111.7 (2)
C3—P1—C1	98.94 (11)	C4—C5—H5A	109.3
C1^i^—P1—C1	92.49 (16)	C6—C5—H5A	109.3
C7—N1—C2	120.8 (2)	C4—C5—H5B	109.3
C7—N1—C1	120.5 (2)	C6—C5—H5B	109.3
C2—N1—C1	111.7 (2)	H5A—C5—H5B	107.9
N1—C1—P1	112.07 (15)	C5^i^—C6—C5	110.8 (3)
N1—C1—H1A	109.2	C5^i^—C6—H6A	109.5
P1—C1—H1A	109.2	C5—C6—H6A	109.5
N1—C1—H1B	109.2	C5^i^—C6—H6B	109.5
P1—C1—H1B	109.2	C5—C6—H6B	109.5
H1A—C1—H1B	107.9	H6A—C6—H6B	108.1
N1—C2—N1^i^	113.9 (3)	N1—C7—C12	122.2 (2)
N1—C2—H2A	108.8	N1—C7—C8	120.4 (2)
N1^i^—C2—H2A	108.8	C12—C7—C8	117.3 (2)
N1—C2—H2B	108.8	C9—C8—C7	121.0 (3)
N1^i^—C2—H2B	108.8	C9—C8—H8	119.5
H2A—C2—H2B	107.7	C7—C8—H8	119.5
C4—C3—C4^i^	109.7 (3)	C8—C9—C10	121.2 (3)
C4—C3—P1	111.07 (17)	C8—C9—H9	119.4
C4^i^—C3—P1	111.07 (17)	C10—C9—H9	119.4
C4—C3—H3A	108.3	C11—C10—C9	118.5 (2)
C4^i^—C3—H3A	108.3	C11—C10—H10	120.8
P1—C3—H3A	108.3	C9—C10—H10	120.8
C5—C4—C3	111.1 (2)	C10—C11—C12	121.3 (3)
C5—C4—H4A	109.4	C10—C11—H11	119.3
C3—C4—H4A	109.4	C12—C11—H11	119.3
C5—C4—H4B	109.4	C11—C12—C7	120.7 (2)
C3—C4—H4B	109.4	C11—C12—H12	119.6
H4A—C4—H4B	108.0	C7—C12—H12	119.6
			
C7—N1—C1—P1	86.3 (2)	C4—C5—C6—C5^i^	54.7 (4)
C2—N1—C1—P1	−64.6 (2)	C2—N1—C7—C12	146.7 (2)
C3—P1—C1—N1	154.91 (18)	C1—N1—C7—C12	−1.5 (3)
C1^i^—P1—C1—N1	55.5 (2)	C2—N1—C7—C8	−37.8 (3)
C7—N1—C2—N1^i^	−86.1 (3)	C1—N1—C7—C8	173.9 (2)
C1—N1—C2—N1^i^	64.6 (3)	N1—C7—C8—C9	−177.0 (2)
C1^i^—P1—C3—C4	165.8 (2)	C12—C7—C8—C9	−1.3 (3)
C1—P1—C3—C4	71.8 (2)	C7—C8—C9—C10	1.8 (4)
C1^i^—P1—C3—C4^i^	−71.8 (2)	C8—C9—C10—C11	−1.1 (4)
C1—P1—C3—C4^i^	−165.8 (2)	C9—C10—C11—C12	−0.1 (4)
C4^i^—C3—C4—C5	56.8 (4)	C10—C11—C12—C7	0.6 (4)
P1—C3—C4—C5	179.9 (2)	N1—C7—C12—C11	175.7 (2)
C3—C4—C5—C6	−56.4 (3)	C8—C7—C12—C11	0.1 (3)

Symmetry code: (i) *x*, −*y*+1/2, *z*.

### 5-Cyclohexyl-1,3-diphenyl-1,3,5-diazaphosphinan-5-one (II) Crystal data

**Table:**

C_21_H_27_N_2_OP	*F*(000) = 380
*M**_r_* = 354.41	*D*_x_ = 1.312 Mg m^−^^3^
Monoclinic, *P*2_1_/*m*	Synchrotron radiation, λ = 0.7288 Å
*a* = 5.2996 (3) Å	Cell parameters from 9912 reflections
*b* = 14.1195 (7) Å	θ = 2.3–29.1°
*c* = 12.0711 (6) Å	µ = 0.17 mm^−^^1^
β = 96.736 (2)°	*T* = 150 K
*V* = 897.02 (8) Å^3^	Plate, colorless
*Z* = 2	0.04 × 0.03 × 0.01 mm

### 5-Cyclohexyl-1,3-diphenyl-1,3,5-diazaphosphinan-5-one (II) Data collection

**Table:**

Bruker D8 diffractometer	2313 independent reflections
Radiation source: synchrotron	1956 reflections with *I* > 2σ(*I*)
Channel-cut Si-<111> monochromator	*R*_int_ = 0.047
Detector resolution: 7.41 pixels mm^-1^	θ_max_ = 29.1°, θ_min_ = 2.3°
combination of ω and φ–scans	*h* = −7→7
Absorption correction: multi-scan (SADABS; Krause *et al.*, 2015)	*k* = −18→18
*T*_min_ = 0.696, *T*_max_ = 0.746	*l* = −16→16
24018 measured reflections	

### 5-Cyclohexyl-1,3-diphenyl-1,3,5-diazaphosphinan-5-one (II) Refinement

**Table:**

Refinement on *F*^2^	Primary atom site location: dual
Least-squares matrix: full	Secondary atom site location: difference Fourier map
*R*[*F*^2^ > 2σ(*F*^2^)] = 0.034	Hydrogen site location: inferred from neighbouring sites
*wR*(*F*^2^) = 0.087	H-atom parameters constrained
*S* = 1.03	*w* = 1/[σ^2^(*F*_o_^2^) + (0.0382*P*)^2^ + 0.3642*P*] where *P* = (*F*_o_^2^ + 2*F*_c_^2^)/3
2313 reflections	(Δ/σ)_max_ < 0.001
121 parameters	Δρ_max_ = 0.32 e Å^−^^3^
0 restraints	Δρ_min_ = −0.37 e Å^−^^3^

### 5-Cyclohexyl-1,3-diphenyl-1,3,5-diazaphosphinan-5-one (II) Special details

**Table:**

Geometry. All esds (except the esd in the dihedral angle between two l.s. planes) are estimated using the full covariance matrix. The cell esds are taken into account individually in the estimation of esds in distances, angles and torsion angles; correlations between esds in cell parameters are only used when they are defined by crystal symmetry. An approximate (isotropic) treatment of cell esds is used for estimating esds involving l.s. planes.

### 5-Cyclohexyl-1,3-diphenyl-1,3,5-diazaphosphinan-5-one (II) Fractional atomic coordinates and isotropic or equivalent isotropic displacement parameters (Å^2^)

**Table:**

	*x*	*y*	*z*	*U*_iso_*/*U*_eq_	
P1	0.44186 (8)	0.750000	0.58754 (3)	0.01761 (12)	
O1	0.1587 (2)	0.750000	0.57920 (10)	0.0247 (3)	
N1	0.54933 (19)	0.66370 (7)	0.39544 (8)	0.0196 (2)	
C1	0.5815 (2)	0.65185 (9)	0.51651 (9)	0.0200 (2)	
H1A	0.765025	0.647569	0.543300	0.024*	
H1B	0.500574	0.591792	0.535671	0.024*	
C2	0.6714 (3)	0.750000	0.36131 (15)	0.0209 (3)	
H2A	0.667862	0.750000	0.279131	0.025*	
H2B	0.851633	0.750000	0.394144	0.025*	
C3	0.6019 (3)	0.750000	0.72862 (13)	0.0197 (3)	
H3A	0.789336	0.750000	0.724639	0.024*	
C4	0.5347 (3)	0.83939 (9)	0.79162 (10)	0.0253 (3)	
H4A	0.586612	0.896269	0.752008	0.030*	
H4B	0.348520	0.842312	0.793260	0.030*	
C5	0.6682 (3)	0.83907 (10)	0.91095 (11)	0.0306 (3)	
H5A	0.854114	0.843194	0.909074	0.037*	
H5B	0.614442	0.895407	0.951021	0.037*	
C6	0.6071 (4)	0.750000	0.97378 (15)	0.0312 (4)	
H6A	0.424649	0.750000	0.984278	0.037*	
H6B	0.706891	0.750000	1.048453	0.037*	
C7	0.3452 (2)	0.62210 (8)	0.32873 (10)	0.0192 (2)	
C8	0.1520 (2)	0.57287 (9)	0.37382 (10)	0.0225 (3)	
H8	0.150975	0.570713	0.452427	0.027*	
C9	−0.0385 (2)	0.52706 (10)	0.30474 (11)	0.0272 (3)	
H9	−0.165646	0.492736	0.337053	0.033*	
C10	−0.0462 (3)	0.53059 (10)	0.18996 (11)	0.0290 (3)	
H10	−0.178353	0.499959	0.143280	0.035*	
C11	0.1428 (3)	0.57973 (10)	0.14460 (11)	0.0292 (3)	
H11	0.139163	0.583094	0.065830	0.035*	
C12	0.3368 (3)	0.62402 (9)	0.21176 (10)	0.0255 (3)	
H12	0.466260	0.656206	0.178536	0.031*	

### 5-Cyclohexyl-1,3-diphenyl-1,3,5-diazaphosphinan-5-one (II) Atomic displacement parameters (Å^2^)

**Table:**

	*U*^11^	*U*^22^	*U*^33^	*U*^12^	*U*^13^	*U*^23^
P1	0.0169 (2)	0.0194 (2)	0.0156 (2)	0.000	−0.00177 (15)	0.000
O1	0.0188 (6)	0.0313 (7)	0.0235 (6)	0.000	−0.0001 (5)	0.000
N1	0.0212 (5)	0.0202 (5)	0.0168 (5)	−0.0009 (4)	−0.0001 (4)	0.0001 (4)
C1	0.0210 (6)	0.0199 (6)	0.0182 (6)	0.0012 (4)	−0.0019 (4)	0.0006 (4)
C2	0.0197 (8)	0.0211 (8)	0.0221 (8)	0.000	0.0032 (6)	0.000
C3	0.0202 (8)	0.0222 (8)	0.0157 (8)	0.000	−0.0023 (6)	0.000
C4	0.0339 (7)	0.0213 (6)	0.0197 (6)	0.0001 (5)	−0.0014 (5)	−0.0011 (5)
C5	0.0452 (8)	0.0265 (7)	0.0189 (6)	−0.0029 (6)	−0.0021 (6)	−0.0038 (5)
C6	0.0448 (12)	0.0324 (10)	0.0160 (8)	0.000	0.0014 (8)	0.000
C7	0.0201 (6)	0.0167 (5)	0.0202 (6)	0.0039 (4)	−0.0007 (4)	−0.0013 (4)
C8	0.0231 (6)	0.0234 (6)	0.0208 (6)	0.0007 (5)	0.0017 (5)	−0.0019 (5)
C9	0.0236 (6)	0.0268 (6)	0.0311 (7)	−0.0026 (5)	0.0024 (5)	−0.0027 (5)
C10	0.0271 (7)	0.0285 (7)	0.0292 (7)	−0.0005 (5)	−0.0062 (5)	−0.0057 (5)
C11	0.0373 (7)	0.0292 (7)	0.0192 (6)	0.0017 (6)	−0.0050 (5)	−0.0006 (5)
C12	0.0302 (7)	0.0254 (6)	0.0205 (6)	−0.0021 (5)	0.0007 (5)	0.0016 (5)

### 5-Cyclohexyl-1,3-diphenyl-1,3,5-diazaphosphinan-5-one (II) Geometric parameters (Å, º)

**Table:**

P1—O1	1.4924 (13)	C5—C6	1.5229 (17)
P1—C3	1.8114 (17)	C5—H5A	0.9900
P1—C1^i^	1.8309 (12)	C5—H5B	0.9900
P1—C1	1.8309 (12)	C6—H6A	0.9900
N1—C7	1.3999 (15)	C6—H6B	0.9900
N1—C1	1.4609 (15)	C7—C8	1.3993 (17)
N1—C2	1.4615 (14)	C7—C12	1.4077 (17)
C1—H1A	0.9900	C8—C9	1.3914 (18)
C1—H1B	0.9900	C8—H8	0.9500
C2—H2A	0.9900	C9—C10	1.3822 (19)
C2—H2B	0.9900	C9—H9	0.9500
C3—C4	1.5369 (15)	C10—C11	1.383 (2)
C3—C4^i^	1.5369 (16)	C10—H10	0.9500
C3—H3A	1.0000	C11—C12	1.3820 (18)
C4—C5	1.5285 (17)	C11—H11	0.9500
C4—H4A	0.9900	C12—H12	0.9500
C4—H4B	0.9900		
			
O1—P1—C3	114.82 (8)	H4A—C4—H4B	108.1
O1—P1—C1^i^	115.28 (5)	C6—C5—C4	111.71 (12)
C3—P1—C1^i^	105.65 (5)	C6—C5—H5A	109.3
O1—P1—C1	115.28 (5)	C4—C5—H5A	109.3
C3—P1—C1	105.65 (5)	C6—C5—H5B	109.3
C1^i^—P1—C1	98.38 (8)	C4—C5—H5B	109.3
C7—N1—C1	121.28 (10)	H5A—C5—H5B	107.9
C7—N1—C2	121.71 (11)	C5—C6—C5^i^	111.35 (16)
C1—N1—C2	111.95 (11)	C5—C6—H6A	109.4
N1—C1—P1	112.06 (8)	C5^i^—C6—H6A	109.4
N1—C1—H1A	109.2	C5—C6—H6B	109.4
P1—C1—H1A	109.2	C5^i^—C6—H6B	109.4
N1—C1—H1B	109.2	H6A—C6—H6B	108.0
P1—C1—H1B	109.2	C8—C7—N1	122.40 (11)
H1A—C1—H1B	107.9	C8—C7—C12	117.47 (11)
N1—C2—N1^i^	112.97 (14)	N1—C7—C12	120.02 (11)
N1—C2—H2A	109.0	C9—C8—C7	120.69 (12)
N1^i^—C2—H2A	109.0	C9—C8—H8	119.7
N1—C2—H2B	109.0	C7—C8—H8	119.7
N1^i^—C2—H2B	109.0	C10—C9—C8	121.19 (12)
H2A—C2—H2B	107.8	C10—C9—H9	119.4
C4—C3—C4^i^	110.42 (14)	C8—C9—H9	119.4
C4—C3—P1	110.80 (8)	C9—C10—C11	118.51 (12)
C4^i^—C3—P1	110.80 (8)	C9—C10—H10	120.7
C4—C3—H3A	108.2	C11—C10—H10	120.7
C4^i^—C3—H3A	108.2	C12—C11—C10	121.22 (12)
P1—C3—H3A	108.2	C12—C11—H11	119.4
C5—C4—C3	110.81 (11)	C10—C11—H11	119.4
C5—C4—H4A	109.5	C11—C12—C7	120.89 (12)
C3—C4—H4A	109.5	C11—C12—H12	119.6
C5—C4—H4B	109.5	C7—C12—H12	119.6
C3—C4—H4B	109.5		
			
C7—N1—C1—P1	−94.67 (11)	C3—C4—C5—C6	−55.80 (17)
C2—N1—C1—P1	60.54 (12)	C4—C5—C6—C5^i^	54.7 (2)
O1—P1—C1—N1	74.33 (10)	C1—N1—C7—C8	4.59 (17)
C3—P1—C1—N1	−157.77 (8)	C2—N1—C7—C8	−148.20 (12)
C1^i^—P1—C1—N1	−48.83 (11)	C1—N1—C7—C12	−171.59 (11)
C7—N1—C2—N1^i^	87.73 (16)	C2—N1—C7—C12	35.61 (17)
C1—N1—C2—N1^i^	−67.35 (16)	N1—C7—C8—C9	−175.64 (11)
O1—P1—C3—C4	−61.46 (10)	C12—C7—C8—C9	0.64 (18)
C1^i^—P1—C3—C4	66.72 (11)	C7—C8—C9—C10	−1.5 (2)
C1—P1—C3—C4	170.35 (9)	C8—C9—C10—C11	1.0 (2)
O1—P1—C3—C4^i^	61.46 (10)	C9—C10—C11—C12	0.4 (2)
C1^i^—P1—C3—C4^i^	−170.35 (9)	C10—C11—C12—C7	−1.3 (2)
C1—P1—C3—C4^i^	−66.72 (11)	C8—C7—C12—C11	0.74 (19)
C4^i^—C3—C4—C5	56.44 (18)	N1—C7—C12—C11	177.11 (12)
P1—C3—C4—C5	179.58 (10)		

Symmetry code: (i) *x*, −*y*+3/2, *z*.

### trans-Dichloridobis(5-cyclohexyl-1,3-diphenyl-1,3,5-diazaphosphinane-κP)nickel(II) (III) Crystal data

**Table:**

[NiCl_2_(C_21_H_27_N_2_P)_2_]	*F*(000) = 1704
*M**_r_* = 806.44	*D*_x_ = 1.349 Mg m^−^^3^
Monoclinic, *C*2/*c*	Synchrotron radiation, λ = 0.7288 Å
*a* = 23.5711 (15) Å	Cell parameters from 9967 reflections
*b* = 10.0062 (6) Å	θ = 2.3–27.8°
*c* = 17.8428 (10) Å	µ = 0.79 mm^−^^1^
β = 109.371 (3)°	*T* = 150 K
*V* = 3970.1 (4) Å^3^	Tablet, orange
*Z* = 4	0.06 × 0.04 × 0.04 mm

### trans-Dichloridobis(5-cyclohexyl-1,3-diphenyl-1,3,5-diazaphosphinane-κP)nickel(II) (III) Data collection

**Table:**

Bruker D8 diffractometer	4383 independent reflections
Radiation source: synchrotron	3341 reflections with *I* > 2σ(*I*)
Channel-cut Si-<111> monochromator	*R*_int_ = 0.103
Detector resolution: 7.41 pixels mm^-1^	θ_max_ = 27.9°, θ_min_ = 1.9°
combination of ω and φ–scans	*h* = −30→29
Absorption correction: multi-scan (SADABS; Krause *et al.*, 2015)	*k* = −12→12
*T*_min_ = 0.690, *T*_max_ = 0.746	*l* = −22→22
36108 measured reflections	

### trans-Dichloridobis(5-cyclohexyl-1,3-diphenyl-1,3,5-diazaphosphinane-κP)nickel(II) (III) Refinement

**Table:**

Refinement on *F*^2^	Primary atom site location: dual
Least-squares matrix: full	Secondary atom site location: difference Fourier map
*R*[*F*^2^ > 2σ(*F*^2^)] = 0.056	Hydrogen site location: inferred from neighbouring sites
*wR*(*F*^2^) = 0.101	H-atom parameters constrained
*S* = 1.07	*w* = 1/[σ^2^(*F*_o_^2^) + 13.1065*P*] where *P* = (*F*_o_^2^ + 2*F*_c_^2^)/3
4383 reflections	(Δ/σ)_max_ < 0.001
232 parameters	Δρ_max_ = 0.37 e Å^−^^3^
0 restraints	Δρ_min_ = −0.41 e Å^−^^3^

### trans-Dichloridobis(5-cyclohexyl-1,3-diphenyl-1,3,5-diazaphosphinane-κP)nickel(II) (III) Special details

**Table:**

Geometry. All esds (except the esd in the dihedral angle between two l.s. planes) are estimated using the full covariance matrix. The cell esds are taken into account individually in the estimation of esds in distances, angles and torsion angles; correlations between esds in cell parameters are only used when they are defined by crystal symmetry. An approximate (isotropic) treatment of cell esds is used for estimating esds involving l.s. planes.

### trans-Dichloridobis(5-cyclohexyl-1,3-diphenyl-1,3,5-diazaphosphinane-κP)nickel(II) (III) Fractional atomic coordinates and isotropic or equivalent isotropic displacement parameters (Å^2^)

**Table:**

	*x*	*y*	*z*	*U*_iso_*/*U*_eq_	
Ni1	0.750000	0.250000	0.500000	0.01964 (15)	
Cl1	0.76558 (4)	0.44883 (8)	0.55189 (4)	0.02871 (19)	
P1	0.70172 (3)	0.33837 (8)	0.38232 (4)	0.01920 (18)	
N1	0.67565 (11)	0.3202 (2)	0.22142 (14)	0.0237 (6)	
N2	0.70331 (11)	0.5407 (2)	0.27898 (14)	0.0248 (6)	
C1	0.70476 (13)	0.2463 (3)	0.29518 (16)	0.0230 (7)	
H1A	0.747278	0.229128	0.300466	0.028*	
H1B	0.684546	0.158821	0.292651	0.028*	
C2	0.70808 (15)	0.4453 (3)	0.22065 (18)	0.0277 (7)	
H2A	0.751070	0.424756	0.230806	0.033*	
H2B	0.691932	0.486095	0.167194	0.033*	
C3	0.73197 (13)	0.4982 (3)	0.36111 (17)	0.0236 (7)	
H3A	0.725876	0.567810	0.397123	0.028*	
H3B	0.775779	0.488852	0.371885	0.028*	
C4	0.62106 (13)	0.3605 (3)	0.36669 (17)	0.0228 (7)	
H4	0.601435	0.400019	0.312796	0.027*	
C5	0.59203 (15)	0.2247 (3)	0.3701 (2)	0.0404 (9)	
H5A	0.594290	0.168635	0.325428	0.048*	
H5B	0.614992	0.179076	0.420194	0.048*	
C6	0.52635 (15)	0.2373 (4)	0.3655 (2)	0.0477 (10)	
H6A	0.502135	0.270773	0.312234	0.057*	
H6B	0.510774	0.148019	0.372609	0.057*	
C7	0.51960 (15)	0.3307 (4)	0.4278 (2)	0.0432 (10)	
H7A	0.540664	0.293261	0.481193	0.052*	
H7B	0.476485	0.340089	0.421717	0.052*	
C8	0.54550 (16)	0.4663 (4)	0.4205 (3)	0.0546 (12)	
H8A	0.541466	0.526066	0.462682	0.066*	
H8B	0.522688	0.506178	0.368461	0.066*	
C9	0.61192 (15)	0.4545 (4)	0.4280 (2)	0.0445 (10)	
H9A	0.627533	0.543993	0.421389	0.053*	
H9B	0.635113	0.421636	0.481755	0.053*	
C10	0.65417 (13)	0.2439 (3)	0.15073 (17)	0.0209 (6)	
C11	0.62063 (14)	0.1279 (3)	0.14937 (19)	0.0269 (7)	
H11	0.614858	0.098394	0.196935	0.032*	
C12	0.59571 (14)	0.0555 (3)	0.08023 (19)	0.0309 (8)	
H12	0.573213	−0.023042	0.080877	0.037*	
C13	0.60329 (16)	0.0964 (3)	0.01029 (19)	0.0343 (8)	
H13	0.585589	0.047754	−0.037527	0.041*	
C14	0.63691 (16)	0.2087 (3)	0.01126 (19)	0.0351 (8)	
H14	0.642740	0.236638	−0.036542	0.042*	
C15	0.66262 (15)	0.2825 (3)	0.07993 (18)	0.0289 (7)	
H15	0.685979	0.359436	0.078846	0.035*	
C16	0.65664 (14)	0.6354 (3)	0.26034 (17)	0.0245 (7)	
C17	0.60640 (15)	0.6278 (3)	0.19134 (19)	0.0350 (8)	
H17	0.601986	0.553800	0.156474	0.042*	
C18	0.56324 (17)	0.7267 (4)	0.1735 (2)	0.0418 (9)	
H18	0.529596	0.720339	0.126153	0.050*	
C19	0.56809 (16)	0.8343 (4)	0.2232 (2)	0.0397 (9)	
H19	0.537958	0.901630	0.210400	0.048*	
C20	0.61703 (15)	0.8435 (3)	0.2915 (2)	0.0327 (8)	
H20	0.620357	0.916983	0.326411	0.039*	
C21	0.66137 (15)	0.7469 (3)	0.30972 (18)	0.0279 (7)	
H21	0.695493	0.755988	0.356299	0.034*	

### trans-Dichloridobis(5-cyclohexyl-1,3-diphenyl-1,3,5-diazaphosphinane-κP)nickel(II) (III) Atomic displacement parameters (Å^2^)

**Table:**

	*U*^11^	*U*^22^	*U*^33^	*U*^12^	*U*^13^	*U*^23^
Ni1	0.0214 (3)	0.0207 (3)	0.0178 (3)	0.0013 (2)	0.0079 (2)	−0.0002 (2)
Cl1	0.0395 (5)	0.0228 (4)	0.0213 (4)	0.0011 (3)	0.0067 (3)	−0.0018 (3)
P1	0.0215 (4)	0.0208 (4)	0.0171 (4)	0.0007 (3)	0.0088 (3)	0.0000 (3)
N1	0.0371 (15)	0.0187 (14)	0.0175 (14)	−0.0042 (11)	0.0123 (12)	0.0003 (11)
N2	0.0368 (15)	0.0219 (14)	0.0195 (14)	−0.0027 (12)	0.0142 (12)	−0.0002 (11)
C1	0.0278 (16)	0.0249 (17)	0.0193 (16)	0.0021 (13)	0.0121 (13)	−0.0008 (13)
C2	0.043 (2)	0.0229 (17)	0.0244 (18)	−0.0051 (14)	0.0212 (15)	−0.0022 (14)
C3	0.0273 (17)	0.0236 (17)	0.0209 (17)	−0.0044 (13)	0.0092 (14)	0.0002 (13)
C4	0.0218 (15)	0.0290 (17)	0.0184 (16)	0.0017 (13)	0.0079 (13)	0.0015 (13)
C5	0.0304 (19)	0.035 (2)	0.062 (3)	−0.0072 (15)	0.0236 (18)	−0.0129 (19)
C6	0.0288 (19)	0.056 (3)	0.066 (3)	−0.0107 (18)	0.0262 (19)	−0.023 (2)
C7	0.0264 (18)	0.069 (3)	0.041 (2)	−0.0039 (18)	0.0200 (17)	−0.009 (2)
C8	0.037 (2)	0.054 (3)	0.083 (3)	0.0025 (19)	0.032 (2)	−0.026 (2)
C9	0.0313 (19)	0.042 (2)	0.068 (3)	−0.0066 (16)	0.0276 (19)	−0.026 (2)
C10	0.0267 (16)	0.0205 (16)	0.0184 (16)	0.0060 (13)	0.0114 (13)	0.0016 (13)
C11	0.0320 (18)	0.0280 (18)	0.0255 (18)	−0.0010 (14)	0.0159 (15)	0.0004 (14)
C12	0.0342 (18)	0.0291 (19)	0.0305 (19)	−0.0038 (15)	0.0121 (16)	−0.0024 (15)
C13	0.052 (2)	0.0273 (19)	0.0194 (18)	0.0018 (16)	0.0057 (16)	−0.0036 (14)
C14	0.061 (2)	0.0277 (19)	0.0213 (18)	0.0039 (17)	0.0195 (17)	0.0033 (15)
C15	0.0402 (19)	0.0260 (18)	0.0233 (18)	0.0002 (14)	0.0145 (15)	0.0016 (14)
C16	0.0343 (18)	0.0240 (17)	0.0199 (17)	−0.0078 (14)	0.0152 (15)	0.0024 (13)
C17	0.045 (2)	0.0298 (19)	0.0245 (19)	−0.0108 (16)	0.0045 (16)	−0.0003 (15)
C18	0.043 (2)	0.037 (2)	0.037 (2)	−0.0030 (17)	0.0030 (18)	0.0116 (18)
C19	0.038 (2)	0.041 (2)	0.045 (2)	0.0043 (17)	0.0205 (19)	0.0166 (18)
C20	0.047 (2)	0.0289 (19)	0.0279 (19)	0.0023 (16)	0.0198 (17)	0.0016 (15)
C21	0.0375 (19)	0.0265 (18)	0.0216 (17)	−0.0017 (15)	0.0122 (15)	0.0006 (14)

### trans-Dichloridobis(5-cyclohexyl-1,3-diphenyl-1,3,5-diazaphosphinane-κP)nickel(II) (III) Geometric parameters (Å, º)

**Table:**

Ni1—Cl1^i^	2.1736 (8)	C7—H7A	0.9900
Ni1—Cl1	2.1736 (8)	C7—H7B	0.9900
Ni1—P1	2.2138 (8)	C8—C9	1.531 (5)
Ni1—P1^i^	2.2138 (8)	C8—H8A	0.9900
P1—C1	1.829 (3)	C8—H8B	0.9900
P1—C3	1.841 (3)	C9—H9A	0.9900
P1—C4	1.841 (3)	C9—H9B	0.9900
N1—C10	1.417 (4)	C10—C15	1.397 (4)
N1—C1	1.467 (4)	C10—C11	1.400 (4)
N1—C2	1.469 (4)	C11—C12	1.383 (4)
N2—C16	1.406 (4)	C11—H11	0.9500
N2—C2	1.444 (4)	C12—C13	1.380 (4)
N2—C3	1.459 (4)	C12—H12	0.9500
C1—H1A	0.9900	C13—C14	1.372 (5)
C1—H1B	0.9900	C13—H13	0.9500
C2—H2A	0.9900	C14—C15	1.387 (4)
C2—H2B	0.9900	C14—H14	0.9500
C3—H3A	0.9900	C15—H15	0.9500
C3—H3B	0.9900	C16—C17	1.399 (4)
C4—C9	1.511 (4)	C16—C21	1.402 (4)
C4—C5	1.532 (4)	C17—C18	1.379 (5)
C4—H4	1.0000	C17—H17	0.9500
C5—C6	1.528 (4)	C18—C19	1.375 (5)
C5—H5A	0.9900	C18—H18	0.9500
C5—H5B	0.9900	C19—C20	1.376 (5)
C6—C7	1.502 (5)	C19—H19	0.9500
C6—H6A	0.9900	C20—C21	1.381 (4)
C6—H6B	0.9900	C20—H20	0.9500
C7—C8	1.511 (5)	C21—H21	0.9500
			
Cl1^i^—Ni1—Cl1	180.0	C6—C7—C8	110.5 (3)
Cl1^i^—Ni1—P1	90.03 (3)	C6—C7—H7A	109.6
Cl1—Ni1—P1	89.97 (3)	C8—C7—H7A	109.6
Cl1^i^—Ni1—P1^i^	89.97 (3)	C6—C7—H7B	109.6
Cl1—Ni1—P1^i^	90.03 (3)	C8—C7—H7B	109.6
P1—Ni1—P1^i^	180.0	H7A—C7—H7B	108.1
C1—P1—C3	97.82 (14)	C7—C8—C9	110.7 (3)
C1—P1—C4	105.16 (14)	C7—C8—H8A	109.5
C3—P1—C4	108.38 (14)	C9—C8—H8A	109.5
C1—P1—Ni1	116.88 (10)	C7—C8—H8B	109.5
C3—P1—Ni1	115.44 (10)	C9—C8—H8B	109.5
C4—P1—Ni1	111.83 (10)	H8A—C8—H8B	108.1
C10—N1—C1	116.7 (2)	C4—C9—C8	111.9 (3)
C10—N1—C2	119.0 (2)	C4—C9—H9A	109.2
C1—N1—C2	110.4 (2)	C8—C9—H9A	109.2
C16—N2—C2	121.1 (3)	C4—C9—H9B	109.2
C16—N2—C3	120.0 (2)	C8—C9—H9B	109.2
C2—N2—C3	114.2 (2)	H9A—C9—H9B	107.9
N1—C1—P1	111.7 (2)	C15—C10—C11	117.6 (3)
N1—C1—H1A	109.3	C15—C10—N1	122.6 (3)
P1—C1—H1A	109.3	C11—C10—N1	119.7 (3)
N1—C1—H1B	109.3	C12—C11—C10	121.4 (3)
P1—C1—H1B	109.3	C12—C11—H11	119.3
H1A—C1—H1B	107.9	C10—C11—H11	119.3
N2—C2—N1	113.0 (2)	C13—C12—C11	120.5 (3)
N2—C2—H2A	109.0	C13—C12—H12	119.8
N1—C2—H2A	109.0	C11—C12—H12	119.8
N2—C2—H2B	109.0	C14—C13—C12	118.6 (3)
N1—C2—H2B	109.0	C14—C13—H13	120.7
H2A—C2—H2B	107.8	C12—C13—H13	120.7
N2—C3—P1	112.2 (2)	C13—C14—C15	122.0 (3)
N2—C3—H3A	109.2	C13—C14—H14	119.0
P1—C3—H3A	109.2	C15—C14—H14	119.0
N2—C3—H3B	109.2	C14—C15—C10	120.0 (3)
P1—C3—H3B	109.2	C14—C15—H15	120.0
H3A—C3—H3B	107.9	C10—C15—H15	120.0
C9—C4—C5	110.5 (3)	C17—C16—C21	117.7 (3)
C9—C4—P1	110.7 (2)	C17—C16—N2	122.6 (3)
C5—C4—P1	109.9 (2)	C21—C16—N2	119.6 (3)
C9—C4—H4	108.6	C18—C17—C16	120.5 (3)
C5—C4—H4	108.6	C18—C17—H17	119.7
P1—C4—H4	108.6	C16—C17—H17	119.7
C6—C5—C4	112.5 (3)	C19—C18—C17	121.1 (3)
C6—C5—H5A	109.1	C19—C18—H18	119.5
C4—C5—H5A	109.1	C17—C18—H18	119.5
C6—C5—H5B	109.1	C18—C19—C20	119.3 (3)
C4—C5—H5B	109.1	C18—C19—H19	120.4
H5A—C5—H5B	107.8	C20—C19—H19	120.4
C7—C6—C5	111.5 (3)	C19—C20—C21	120.6 (3)
C7—C6—H6A	109.3	C19—C20—H20	119.7
C5—C6—H6A	109.3	C21—C20—H20	119.7
C7—C6—H6B	109.3	C20—C21—C16	120.8 (3)
C5—C6—H6B	109.3	C20—C21—H21	119.6
H6A—C6—H6B	108.0	C16—C21—H21	119.6
			
C10—N1—C1—P1	−156.1 (2)	P1—C4—C9—C8	−175.5 (3)
C2—N1—C1—P1	63.9 (3)	C7—C8—C9—C4	57.2 (5)
C3—P1—C1—N1	−51.3 (2)	C1—N1—C10—C15	−136.3 (3)
C4—P1—C1—N1	60.2 (2)	C2—N1—C10—C15	0.1 (4)
Ni1—P1—C1—N1	−175.08 (16)	C1—N1—C10—C11	46.7 (4)
C16—N2—C2—N1	−90.4 (3)	C2—N1—C10—C11	−176.8 (3)
C3—N2—C2—N1	65.4 (3)	C15—C10—C11—C12	−1.2 (4)
C10—N1—C2—N2	153.2 (3)	N1—C10—C11—C12	175.9 (3)
C1—N1—C2—N2	−67.9 (3)	C10—C11—C12—C13	−0.2 (5)
C16—N2—C3—P1	98.6 (3)	C11—C12—C13—C14	1.2 (5)
C2—N2—C3—P1	−57.5 (3)	C12—C13—C14—C15	−0.8 (5)
C1—P1—C3—N2	47.2 (2)	C13—C14—C15—C10	−0.6 (5)
C4—P1—C3—N2	−61.6 (2)	C11—C10—C15—C14	1.6 (4)
Ni1—P1—C3—N2	172.05 (16)	N1—C10—C15—C14	−175.4 (3)
C1—P1—C4—C9	−170.9 (2)	C2—N2—C16—C17	12.6 (4)
C3—P1—C4—C9	−67.1 (3)	C3—N2—C16—C17	−141.8 (3)
Ni1—P1—C4—C9	61.3 (3)	C2—N2—C16—C21	−163.6 (3)
C1—P1—C4—C5	66.7 (2)	C3—N2—C16—C21	41.9 (4)
C3—P1—C4—C5	170.5 (2)	C21—C16—C17—C18	−0.4 (5)
Ni1—P1—C4—C5	−61.1 (2)	N2—C16—C17—C18	−176.7 (3)
C9—C4—C5—C6	52.1 (4)	C16—C17—C18—C19	−0.6 (5)
P1—C4—C5—C6	174.5 (3)	C17—C18—C19—C20	0.3 (5)
C4—C5—C6—C7	−54.1 (4)	C18—C19—C20—C21	0.9 (5)
C5—C6—C7—C8	56.6 (4)	C19—C20—C21—C16	−1.9 (5)
C6—C7—C8—C9	−58.0 (4)	C17—C16—C21—C20	1.6 (4)
C5—C4—C9—C8	−53.6 (4)	N2—C16—C21—C20	178.0 (3)

Symmetry code: (i) −*x*+3/2, −*y*+1/2, −*z*+1.
